# Use of complementary and alternative medicines in haemodialysis patients: a cross-sectional study from Palestine

**DOI:** 10.1186/s12906-016-1196-8

**Published:** 2016-07-11

**Authors:** Sa’ed H. Zyoud, Samah W. Al-Jabi, Waleed M Sweileh, Ghada H. Tabeeb, Nora A. Ayaseh, Mayas N. Sawafta, Razan L. Khdeir, Diana O. Mezyed, Dala N. Daraghmeh, Rahmat Awang

**Affiliations:** Poison Control and Drug Information Center (PCDIC), College of Medicine and Health Sciences, An-Najah National University, Nablus, 44839 Palestine; Department of Clinical and Community Pharmacy, College of Medicine and Health Sciences, An-Najah National University, Nablus, 44839 Palestine; WHO Collaborating Centre for Drug Information, National Poison Centre, Universiti Sains Malaysia (USM), Penang, 11800 Malaysia; Department of Pharmacology and Toxicology, College of Medicine and Health Sciences, An-Najah National University, Nablus, 44839 Palestine; PharmD program, College of Medicine and Health Sciences, An-Najah National University, Nablus, Palestine

**Keywords:** Haemodialysis, End-stage renal disease, Complementary and alternative medicine, Herbal medicine, Palestine

## Abstract

**Background:**

Complementary and alternative medicine (CAM), and herbal therapies, are accepted worldwide, and have been important from medical, sociological and economic perspectives, among haemodialysis (HD) patients. The primary aim of this study was to evaluate the use of CAM among patients with end-stage renal disease (ESRD) who are undergoing HD.

**Methods:**

Face-to-face interviews of patients with ESRD undergoing HD from ten outpatient renal departments at a national level in Palestine were conducted from June 2014 to January 2015. A survey questionnaire, which included questions on socio-demographic and clinical characteristics, and on the CAM therapies that were used, was administered.

**Results:**

Out of 267 patients interviewed, 172 patients used at least one type of CAM in the last month prior to the interview, and thus the utilisation rate was 64.4 %. Forty one (15.4 %) patients reported using one type of CAMs, while 18.7 % used two different CAMs and 30.3 % used more than two types of CAMs for their health status. Of the patients who used CAM, herbal therapies were used most often (43.5 %), followed by honey (35.6 %), diet (22.8 %), and exorcism in Islam (16.9 %). The herbal therapies mentioned most often were *Nigella sativa* L. (18.7 %), followed by *Salvia officinalis* L. (16.9 %), and *Pimpinella anisum* L. (10.5 %).

**Conclusions:**

In conclusion, the prevalence of CAM is relatively high in the selected population. Most patients used biological therapies such as herbal remedies, thus highlighting a greater need for patient education regarding CAM therapies and possible herb-drug interactions. Health care providers must be aware of the potential benefits and risks related to CAM use. There is a need for more clinical research pertaining to CAM to reach stronger evidence regarding potential benefits and risks related to CAM use.

**Electronic supplementary material:**

The online version of this article (doi:10.1186/s12906-016-1196-8) contains supplementary material, which is available to authorized users.

## Background

Haemodialysis (HD) is the first-line treatment for patients with end-stage renal disease (ESRD), designed to replace kidney function, and it is the most extensively used technique worldwide [1–3]. Complementary and alternative medicine (CAM), or herbal therapies, are extremely popular worldwide and have been important among HD patients from medical, sociological and economic perspectives [4–6]. CAM may provide new therapeutic opportunities for HD patients with the target of enhancing quality of life and improving symptoms [7–9].

The most widely used CAM therapies include biologically based therapies (e.g. herbal) and mind-body medicine (e.g. yoga, spiritual healing/prayer, meditation, and hypnosis). Despite the ubiquitous use of CAM at a global level in a broad range of illnesses or populations, there is limited data concerning the use of CAM or herbal therapies in patients with ESRD [7, 8, 10–12]. To our knowledge, there were several studies conducted on CAM, considering aspects other than ESRD type, such as CAM among Palestinian hypertensive patients [13], diabetes mellitus patients [14], cancer patients [15], or the general public [16]. There were also several studies conducted on herbal therapies that considered aspects other than ESRD type, such as herbal therapies among cancer patients [17], university students [18], geriatric patients [19], or in pregnancy [20, 21]. An extensive search did not reveal information concerning the use of CAM or herbal among HD patients in the Arab Middle East where medicinal herbs, or some types of CAM such as honey, prayers, reading holy books, Al-Hijama, fasting, or exorcism in Islam are an integral part of the culture and religion [22–27]. This study was performed to determine the prevalence and types of CAM and herbal therapies used among Palestinian patients with HD. The present study aims to contribute to this growing area of research by helping in plan the interventions needed to improve the self-use of CAM or herbal therapies, and by identifying the most commonly used CAM or herbal therapies which may form a data-base for further research. Understanding patterns of CAM therapies used in HD patients will not only help healthcare providers to provide more informed clinical care but also assist policymakers in creating the appropriate frameworks for future policy and direct and encourage researchers to conduct clinical research pertaining to CAM to reach stronger evidence regarding potential benefits and risks related to CAM use.

## Methods

### Study design and setting

Data in this study was obtained from a cross-sectional study carried out during the period: June 2014 to January 2015. Participants were recruit from ten different settings (Abu Al hasan Al Kassem Hospital, Al Hussein Hospital, Alia Hospital, An-najah National University teaching Hospital, Darwish Nazal Hospital, Jericho Government Hospital, Khalil Suliman Hospital, Ramallah’s Sons Wing Hospital, Thabit Thabit Hospital, and Yasser Arafat Hospital) representing government and non-government sectors in West-Bank, Palestine to include a broad spectrum of participant characteristics [28].

### Study population

Consecutive dialysis patients from 10 outpatient renal departments with associated dialysis units were included. Information regarding number and geographical distribution of dialysis units in West-Bank as well as the total number of HD patients (740) at the time of study were obtained from the Palestinian Ministry of Health and Palestinian Central Bureau of Statistics [29].

### Sample size and sampling techniques

Raosoft sample size calculator, a web-based calculator, was used to estimate the required sample size assuming that 50 % of the HD patients used CAM for their health status, margin of error set at 0.05, and assuming a confidence level of 95 % [30]. The required sample size was calculated to be 254 HD patients, which was increased to 277 for more accuracy, to account for unusable questionnaires and potential non-responses, and to increase the generalizability of the obtained results. Participants who met the following criteria were included in the study: (1) patients younger than 18; (2) confirmed diagnosis of ESRD; and (3) on regular HD therapy for at least 6 months. Those patients who were seriously ill or had major psychiatric disorders at the time of study were excluded.

### Research instrument

A quantitative survey instrument was formed based on questionnaires from previous studies [7, 8, 10–12]. The questionnaires were prepared and evaluated for content validity by a group of experts in the fields of nephrology, alternative medicine, clinical pharmacy and biostatisticians. The clarity and readability of the questionnaire was pre-tested in a pilot study of 16 patients. The results of the pilot sample were not included in the final analysis. Feedback from the participants was used to modify and adjust the questionnaire to reach the final version of the study tool. An additional file was provided to show detailed description of the study both in English and Arabic (Additional file 1).

The questionnaire consisted of four parts. The first part was about the socio-demographic characteristics of the participants, and recorded details of age, gender, marital status, residency, educational level, body mass index (BMI), family monthly income, smoking status, and occupational status. The second part was about the clinical characteristics of the participants, and recorded the duration of disease in months, duration of dialysis sessions in hours, number of dialysis per week, number of chronic diseases, and number of medications for chronic use. The third part of the questionnaire focused on the regular consumption of CAM and participants were asked to identify what they had used in the last month before the survey. CAM therapies were categorized in a list as follows: (1) Alternative Medical Systems such as acupuncture; (2) Biologically Based Therapies such as folk medicine, vitamins, or other types of herbal products; (3) Manipulative and Body-Based Methods such as massage or physical therapies (e.g. heat and cold, or rehabilitation strategies); and (4) Mind-Body Medicine such as meditation, hypnosis, walking, or music therapy [31–38]. Exorcism in Islam (ruqya) was combined with Mind-Body Medicine, to mimic previous studies [33, 39, 40]. The last part of the questionnaire was designed to determine the types of herbal therapies that HD patients have used in self-therapy practices. HD patients were requested to provide the native name of the herb that they used as self-therapy. This study included all herbal remedies or other CAM which was used only for improving or curing health conditions during the dialysis care period as CAM.

### Statistical analysis

All statistical analyses were conducted using the Statistical Software Package for the Social Sciences, version 15 for Windows (SPSS, Chicago, IL). For descriptive statistics, means, standard deviations (SD), and medians interquartile range [IQR]), frequencies, and percentages were computed. Pearson Chi-Square test or Fisher’s exact test was used for comparative analysis. A value of *P* < 0.05 was considered significant.

## Results

A total of 267 patients (139 males, 128 females; mean age 53.3 ± 16.2 years) receiving HD were recruited for the study. The socio-demographic data of the study participants is listed in Table 1. The majority of patients (73.4 %) had hypertension. Approximately two thirds (67 %) had multiple chronic diseases, and 172 (64.4 %) were on six or more chronic medications. Around two thirds of patients (76.4 %) were dialyzed three times weekly, 188 patients (70.4 %) stayed on dialysis more than three hours, and the median duration of HD was 2 (interquartile range: 1–5 years). Demographic and clinical characteristics of the participants are shown in Table 1.

**Table 1:** Socio-demographic and clinical characteristics of the study population (*n* = 267) and their association with CAM use

Variable	Overall N (%) *N* =267	CAM users *N* (%) *N* = 172	Non-CAM users *N* (%) *N* = 95	*P* value
Age category (year)				0.093
< 40	58 (21.7)	42 (24.4)	16 (16.8)	
40–59	97 (36.3)	66 (38.4)	31 (32.6)	
≥ 60	112 (41.9)	64 (37.2)	48 (50.5)	
Gender				0.899
Male	139 (52.1)	89 (51.7)	50 (52.6)	
Female	128 (47.9)	83 (48.3)	45 (47.4)	
BMI				0.323
Underweight	24 (9)	16 (9.4)	8 (9.3)	
Normal	97 (36.3)	71 (41.8)	26 (30.2)	
Overweight	27 (28.1)	44 (25.9)	28 (32.6)	
Obese	63 (23.6)	39 (22.9)	24 (27.9)	
Education				0.112
Not educated	40 (15.0)	21(12.2)	19 (15)	
Elementary school (primary)	71 (26.6)	42 (24.4)	29 (30.5)	
High school (secondary school)	102 (38.2)	69 (40.1)	33 (34.7)	
University	54 (20.2)	40 (23.3)	14 (14.7)	
Income				0.006
Moderate to high (≥2000 NIS)	100 (37.5)	75 (43.6)	25 (26.3)	
Low (<2000 NIS)	167 (62.5)	97(56.4)	70 (73.7)	
Living				0.559
City	84 (31.5)	58 (33.7)	26 (27.4)	
Village	161 (60.3)	100 (58.1)	61 (64.2)	
Palestinian refugee camps	22 (8.2)	14 (8.1)	8 (8.4)	
Marital Status				0.404
Married	188 (70.4)	118 (68.6)	70 (73.7)	
Not married	79 (29.6)	54 (20.2)	25 (26.3)	
Occupation				0.706
Unemployed	232 (86.9)	148 (86.0)	84 (88.4)	
Employed	35 (13.1)	24 (14.0)	11 (11.6)	
Duration of dialysis (month)				0.343
< 12	58 (21.7)	40 (23.3)	18 (18.9)	
12–49	119(44.6)	71 (41.3)	48 (50.5)	
≥ 50	90 (33.7)	61 (35.5)	29 (30.5)	
Number of dialysis sessions per week				<0.001
≤ 2	26 (9.7)	14 (8.1)	12 (12.6)	
3	204 (76.4)	144 (83.7)	60 (63.2)	
≥ 4	37 (13.9)	14 (8.1)	23 (24.2)	
Dialysis in a day (hours)				0.066
≤ 3	79 (29.6)	43(25.0)	36 (37.9)	
> 3- < 4	119 (44.6)	84 (48.8)	35 (36.8)	
≥ 4	69 (25.8)	45 (26.2)	24 (25.3)	
Transplantation history				0.830
Yes	26 (9.7)	16 (9.3)	10 (10.5)	
No	241 (90.3)	156 (90.7)	85 (89.5)	
Total chronic co-morbid disease				0.959
Non	26 (9.7)	18 (10.5)	8 (8.4)	
1	62 (23.2)	40 (23.3)	22 (23.2)	
2	63 (23.6)	40 (23.3)	23 (24.2)	
≥ 3	116 (43.4)	74 (43.0)	42 (44.2)	
Presence of diabetes mellitus				0.091
Yes	122 (45.7)	72 (59.0)	50 (52.6)	
No	145 (54.3)	100 (58.1)	45 (47.4)	
Presence of hypertension				0.940
Yes	196 (73.4)	126 (73.3)	70 (73.7)	
No	71 (26.6)	46 (26.7)	25 (26.3)	
Chronic medication (per day)				0.393
< 6	95 (35.6)	58 (33.7)	37 (38.9)	
≥ 6	172 (64.4)	114 (66.3)	58 (61.1)	

Abbreviations: *BMI* body mass index, *CAM* Complementary and alternative medicine, *NIS* New Israeli Shekel

One hundred-seventy two of the patients (64.4 %) reported the use of one or more type of CAM therapy. A total of 19 different CAMs were reported by HD patients (Table 2). A total of 486 CAM episodes were reported by all HD patients, giving an average utilisation rate of 1.8 ± 1.9. The CAM users among the dialysis patients had used a median of one CAM, and a maximum of nine types. Forty one (15.4 %) patients reported using one type of CAM, while 18.7 % used two different CAMs and 30.3 % used more than two types of CAM for their health. Of the patients who used CAM, herbal therapy was used most often (43.5 %), followed by honey (35.6 %), diet (22.8 %), and exorcism in Islam (16.9 %); (Table 2). CAM use was distributed throughout the population in regards to the socio-demographic and clinical characteristics in Table 1. A comparison of the characteristics of users and nonusers of CAM showed that there was no significant difference (*p*-value > 0.05) in all socio-demographics and clinical characteristics except in their income, and the number of dialysis sessions per week which showed a significant difference (*p*-value < 0.05).

**Table 2:** Type of complementary and alternative medicine therapies used by the patients

CAM type	Frequency (%)^a^
Biologically Based Therapies	161 (60.3)
Herbal therapy	116 (43.5)
Honey	95 (35.6)
Diet therapy	61 (22.8)
Folk medicine	39 (14.6)
Manipulative and Body-Based Methods	39 (14.6)
Massage	26 (9.7)
Physiotherapist	23 (8.6)
Mind-Body Medicine	76 (25.1)
Exorcism in Islam (ruqya)	45 (16.9)
Deep breathing	18 (6.7)
Cupping	16 (6)
Relaxation	16 (6)
Exercises	7 (2.6)
Meditation	6 (2.2)
Walking	5 (1.9)
Yoga	3 (1.1)
Hypnosis	2 (0.7)
Music	1 (0.4)
Applaud Islamist	1 (0.4)
Silence	1 (0.4)
Alternative Medical Systems	5 (1.9)
Acupuncture	5 (1.9)

Abbreviation: *CAM* Complementary and alternative medicine

^a^Column total exceeds 100 % as data are overlapping because participants used more than one type of CAM therapy

A total of 20 different herbal therapies were reported by HD patients (Table 3). Herbal therapies mentioned most often were *Nigella sativa* L. (18.7 %), followed by *Salvia officinalis* L. (16.9 %), and *Pimpinella anisum* L. (10.5 %); (Table 3). Fifty two (19.5 %) patients reported using one type of herbal therapy, 10.1 % reported using two types of herbal therapy, and 13.9 % reported using more than two types of herbal therapies for their health.

**Table 3:** Distribution of herbal supplements

Herbs	Frequency (%)
Black cumin seed (*Nigella sativa* L.)	50 (18.7)
Sage (*Salvia officinalis* L.)	45 (16.9)
Anise (*Pimpinella anisum* L.)	28 (10.5)
Chamomile (*Matricaria chamomilla* L.)	28 (10.5)
Senna (*Senna alexandrina* Mill.)	23 (8.6)
Gum Arabic (*Acacia senegal* (L.) Willd)	22 (8.2)
Spearmint (*Mentha spicata* L.)	13 (4.9)
Parsley (*Petroselinum crispum* (Mill.) Fuss)	12 (4.5)
Fenugreek (*Trigonella foenum-graecum* L.)	12 (4.5)
Barley water (*Hordeum vulgare* L.)	9 (3.4)
Ginger (*Zingiber officinale* Roscoe)	2 (0.7)
Green Tea (*Camellia sinensis* (L.) Kuntze)	2 (0.7)
Large-leaved lime *(Tilia platyphyllos* Scop.)	2 (0.7)
Ivy (*Hedera helix* L.)	1 (0.4)
Ginseng (*Panax ginseng* C.A. Mey)	1 (0.4)
Hawthorn (*Crataegus pinnatifida* Bunge)	1 (0.4)
Caraway (*Carum carvi* L.)	1 (0.4)
Olive leaves (*Olea europaea* L.)	1 (0.4)
Cinnamon (*Cinnamomum verum* J. Presl)	1 (0.4)
Verbena (*Verbena officinalis* L.)	1 (0.4)

## Discussion

This cross-sectional study documents the utilisation pattern of CAMs among HD in 10 haemodialysis centres in West Bank, Palestine. The participants are considered representative of patients with ESRD in Palestine since the haemodialysis centres are located in all districts of Palestine. The use of CAM has been reported among HD patients at a global level [7, 8, 10–12], however, the current study is the first to assess the use of CAM among a sample of HD patients in Palestine.

Many of the patients that we studied reported the use of certain types of CAM therapies in their health self-management. These therapies were mainly home herbal remedies. Around two thirds of HD patients in our study reported the use of one, or more than one, type of CAM therapy, which is in agreement with other published studies among HD or other chronic diseases [8, 10, 41, 42]. Use of CAM or herbal remedies among Palestinian people and Arab or Muslim populations in general is driven by culture, history, sometimes religion, and sometimes by herbalists spread all over the country rather by advice from healthcare providers [22–27, 43, 44]. Based on this, the investigators did address the question “who recommends these herbs to HD patients” because of the many possible confounding factors. Furthermore, none of the herbal remedies used by HD patients are present in the essential drug list of the Palestinian ministry of health and are not covered by health insurance which made their use to be outside the context of prescribed medicines [45, 46].

It is noteworthy that the consumption of herbal remedies reported in our study was more prevalent in dialysis patients than that in other published data [10, 11]. Herbal remedies were of interest in this study because they are usually considered intrinsically safe and beneficial, and are frequently consumed in large quantities by ESRD patients [10, 11, 47–49]. *Nigella sativa* L.*, and Salvia officinalis* L. were among the most common herb reported in the current study as well, which is interesting because extensive research studies on *Nigella sativa* L.*, and Salvia officinalis* L. have indicated a renoprotective effects of these herbs [50–52].

However, because herbal constituents and bioactive phytochemical compounds might interact with patient’s medications, their use is not always safe for HD patients [53–57]. The use of herbal medicines in HD patients seems to bear more risk compared tothe general population [11]. It is mainly due to accumulation of toxic material of herbal remedies in patients with kidney malfunction. Toxicity of herbal therapies may be caused by the different active constituents; or contaminants, or potential interactions with other herbs and drugs [58]. More recent studies demonstrated that herbal medicines were associated with enhanced ESRD risk in patients with CKD [59, 60]. Senna, chamomile, and fenugreek were among the most common herbs reported in the current study as well, which is interesting that senna can lead to electrolyte imbalance especially hypokalemia [61]. Chamomile and fenugreek also causes nephrotoxicities [62, 63].

Patients with renal problem are at risk of herb-drug interactions through different mechanisms (e.g. triggered activity changes of cytochrome *P*-450 isoenzyme metabolism and drug transport proteins for many drugs such as antihypertensives, anticoagulants, antidiabetics) [55]. Herb-drug interactions may often be unobserved because healthcare providers are not well-informed about potential herbal-drug interactions occurring in HD patients [54, 55, 64, 65]. Renal impairment exposes patients to the risks of herbal remedies such as electrolyte imbalances [11, 66]. Conversely, herbal remedies used by HD patients may have numerous possible benefits such as decreasing cutaneous pruritus, oxidative stress status, muscle cramps, and dialysis frequency [67, 68] or reducing proteinuria and increasing serum albumin and haemoglobin [69].

Mind-body practices such as yoga, meditation, or progressive relaxation, were the least common CAM type recognised by the participants. However, exorcism was the most common CAM type recognized by the participants. A survey of patients with ESRD in the USA reported that 42 % used mind-body practices [8]. Several studies have demonstrated that mind-body practices may be beneficial for patients with ESRD [70, 71]. Mind–body medicine, such as relaxation, or exorcism often involves inexpensive self-care–based actions and appears to have minimal side effects, risks, or interactions with conventional treatment [7]. Exorcism in Islam (ruqya) is frequently used for healing in the Muslim world [33, 39, 40].

The most important implication of the current study is that in the past decade there has been a remarkable increase in the use of CAM therapies in HD patients. Healthcare providers thus need to be fully aware of the commonly used CAM therapies in their culture, and provide the correct information about the benefits or risks related to CAM therapies that may be used by HD patients [57, 65]. It is essential for healthcare providers to be familiar with the evidence-based medicine related to herbal remedies for HD patients. Evidence is also required to evaluate CAM therapies that may be not harmful most of the time (e.g. prayer, or relaxation), compared with those that may potentially be harmful (e.g. herbal therapy). The current study provides information about the different types of CAM used by HD patients. This information should be helpful to healthcare providers in identifying patients who should be given focused education about the potential benefits and risks of unproved therapies for HD patients [57, 64, 65].

The key strengths of this study are that it includes a multi-district sample; is a national survey with a high response rate, and a large sample size. This study has some limitations worth mentioning: it is limited by its cross-sectional design, and the results are subject to recall bias regarding CAM use. The convenience sampling method (non-random) is considered as source of bias since it will produce a non representative sample. Another limitation is that this study only assessed HD patients’ practises towards CAM and did not survey physicians’ attitude or other health care providers who also provide medical care to HD patients.

## Conclusions

In conclusion, the prevalence of CAM is relatively high in the selected population. Most patients used biologically-based therapies such as herbal remedies, thus highlighting a greater need for patient education regarding CAM therapies and their possible herb-drug interactions. Health care providers must be aware of the potential benefits and risks related to CAM use. There is a need for more clinical research pertaining to CAM to reach stronger evidence regarding potential benefits and risks related to CAM use.

## Abbreviations

BMI, body mass index; CAM, Complementary and alternative medicine; CKD, Chronic kidney disease; ESRD, end-stage renal disease; HD, haemodialysis; IQR, interquartile range; IRB, institutional review board; SD, standard deviation

## Additional file

Additional file 1: Study questionnaires. This is the final version of the English  and Arabic version that was used to evaluate the use of complementary and alternative medicine among patients with end-stage renal disease who are undergoing haemodialysis. (DOC 74 kb)
