# Supplementary material for: Use of complementary and alternative medicines in haemodialysis patients: a cross-sectional study from Palestine
Source: BMC Complement Altern Med. 2016 Jul 11;16:204. doi: 10.1186/s12906-016-1196-8 (PMC4940841; doi:10.1186/s12906-016-1196-8)
Supplement: Additional file 1: — Study questionnaires. This is the final version of the English and Arabic version that was used to evaluate the use of complementary and alternative medicine among patients with end-stage renal disease who are undergoing haemodialysis. (DOC 74 kb) [file 12906_2016_1196_MOESM1_ESM.doc]

**Additional file 1.** **Study questionnaires. This is the final version of the and Arabic version that was used to evaluate the use of complementary and alternative medicine among patients with end-stage renal disease who are undergoing haemodialysis.**

**English Version**

Dear Participant,

The purpose of this questionnaire is to determine the pattern and types of alternative and complementary medicine among dialysis patients, so please answer the following questions.

Note that the information will remain secret and will be used only for the purposes of scientific research.

Thank you.

**A. Patient demographic characteristics**

**A.1 Age:** _______________ years

**A.2 Gender:** □ Male □ Female

**A.3 Weight:** _______ Kg

**A.4 Height:** _________ cm

**A. 5 Locality:**

□ Urban □ Rural □ Palestinian refugees camps

**A. 6 Current residence:**

□ Live alone □ live with family □ other: _______________

**A.7 Level of education:**

□ illiterate □ Primary □ Secondary □ Tawjihi exam

□ Diploma □ University □ Higher education

**A.8 Marital Status:**

□ Married □ Single □ Divorced □ Widowed

**A.9 Employment status**

□ Employed □ Unemployed □ House wife

**A.10 Monthly income:**

□ Low (less than 2000 NIS) □ Moderate (between 2000-5000 NIS) □ High (more than 500 NIS)

**B. History, com-morbid disease and medications**

**B.1 Number of years since you begin dialysis: ______________________.**

**B.2 Number of dialysis per week**

□ two days or less □ three days □ four days □five days □ more ____________

**B.3 Duration of dialysis session (hour): ___________________**

**B.4 Have you ever undergone a kidney transplant**

□ Yes □ No

**B.5 Do you know what is the cause of your illness**

□ Yes , Indicate _______________ □ No

**B.6: Co-morbidities:**

□ Hypertension □ Diabetes mellitus

□ Dyslipidemia □ Arthritis

□Heart failure □ Stroke

□ systemic lupus erythematosus □ Chronic lung disease

□ Ischemic heart disease □ Others : …………………….

**B.7: Medications**

| **Drug name** | **Drug name** | **Drug name** |
| --- | --- | --- |
| 1. | 4. | 7. |
| 2. | 5. | 8. |
| 3. | 6. | 9. |
| Others…. |  |  |

**B.4: Smoking**

□ Current smoking (number of cigarettes you smoke per a day: ____________)

□ Previous smoker but noncurrent smoking

□ Non smoker

**C. complementary and alternative medicine**

**C.1 Have you ever used any type of alternative or complementary medicine**

□ Yes □ No

**C.2 If your answer is "Yes" in the previous question, please indicate the type of treatment that you used in the previous month**

□ Massage □ Physiotherapy □ Acupuncture

□ Hypnotism □ Folk medicine □ Music

□ Applaud Islamist □ Other: ____________

**C.3 Have you ever used any type of prophetic medicine that you used in the previous month?**

□ Yes □ No

**C.4 If your answer is "Yes" in the previous question, please indicate the type of prophetic medicine that you used in the previous month**

□ Exorcism in Islam (ruqya) □ Cupping □ Honey

□ Black cumin seed (*Nigella sativa L.*) □ Diet □ Other: ____

**C.5 Have you ever used any type of dietary supplements**

□ Yes □ No

**C.6 If your answer is "Yes" in the previous question, please indicate the type of dietary supplements that you used in the previous month**

___________________________

**C.7 Have you ever used any type of medicinal herbs in the treatment of your health condition**

□ Yes □ No

**C.8 If your answer is "Yes" in the previous question, please indicate the type of medicinal herbs that you used in the previous month**

___________________________

**C.9 Have you ever used any type of kind of mind-body techniques**

□ Yes □ No

**C.10 If your answer is "Yes" in the previous question, please indicate the type of treatment that you used in the previous month**

□ meditation □ relaxation

□ yoga □ deep breathing exercises

□ energy exercises □ Other: ____________

**Arabic version**

**عزيزي المشارك، إن الغرض من هذه الاستمارة هو تحديد نمط وانواع الطب البديل والتكميلي عند مرضى غسيل الكلى ، لذا نرجو منك الإجابة عن الأسئلة التالية ، علما بأن المعلومات التي ستدلي بها ستظل سرية وتستخدم فقط لأغراض البحث العلمي، وشكرا لكم مقدما.**

***ملاحظة: الاستمارة مكتوبة في صيغة المذكر إلا أنها موجهة لكلا الجنسين على حد سواء***

***تتكون هذه الاستمارة من ثلاثة أقسام* :**

**القسم الأول*:***

1-**العمر** ...................

2**- الجنس**: ❑ ذكر ❑ انثى

3- الطول : ...............................

4- الوزن .......................................

5- **مكان الإقامة** :❑ مخيم ❑ قرية ❑ مدينة

6- إقامتك الحالية : ❑ أعيش وحدي ❑ أعيش مع عائلتي ❑ غير ذلك ..............................

7-**ما المستوى التعليمي لديك** : ❑ غير دارس❑ ابتدائي ❑ إعدادي ❑ثانوية عامة ( توجيهي )

❑ كلية ( دبلوم ) ❑ جامعي ( بكالوريوس ) ❑ دراسات عليا

8**- الحالة الإجتماعية:** ❑ أعزب ❑متزوج ❑ مطلق ❑ أرمل

9**- ما نوع عملك ؟** ❑ موظف ❑ غير موظف ❑ ربة منزل

**10- كم يبلغ معدل الدخل الشهري للعائلة ؟**

❑ أقل من 2000 شيقل ❑ 2000-5000 شيقل ❑ أكثر من 5000 شيقل

==============================================================

**القسم الثاني :**

**11- كم من الوقت مر على بداية عملية غسيل الكلى .........................................**

12- **كم يوماً بالأسبوع تخضع لعملية غسيل الكلى :**

❑يومان أو أقل ❑ ثلاثة أيام

❑ أربعة أيام ❑ خمسة أيام ❑ أكثر من ذلك، حدد ................................

13**- كم ساعة تستغرق عملية الغسيل في كل مرة** ............................

14- **هل سبق وقمت بعملية زراعة الكلى:** ❑ نعم ❑لا

15- هل تعرف ما هو سبب مرضك؟ ..........................................................................................

16- هل لديك أمراض أخرى❑ سكري ❑ ضغط الدم ❑ التهاب المفاصل ❑الذئبة الحمراء الجهازية

❑ ذبحة صدرية ❑ جلطة دماغية ❑ قصور في عضلة القلب ❑ مرض رئوي مزمن

❑غيرذلك، اذكرها ...................................................................................

17- **ما هي الأدوية التي تتناولها**

1............................... 2-...........................3-......................................

4...............................5.............................6........................................

7...............................8.............................9........................................

غير ذلك.................................................................................................

18- هل أنت مدخن؟ ❑ نعم ❑ لا

19- إذا كانت إجابتك عن السؤال السابق بنعم .. فكم عدد السجائر التي تدخنها يومياً ؟ .................................

**القسم الثالث (اسئلة تتعلق بالطب البديل والتكميلي)**

1- هل سبق واستخدمت أي نوع من أنواع الطب البديل أو المكمل ؟ ❑ نعم ❑ لا

- إذا كانت إجابتك عن السؤال السابق بـ ( نعم ), اذكر نوع العلاج الذي استخدمته خلال الشهر السابق

❑ التدليك ❑ الوخز بالابر ❑العلاج الطبيعي ❑ التنويم المغناطيسي ❑ الطب الشعبي

❑ سماع الموسيقى ❑ سماع النشيد الاسلامي

غير ذلك، اذكره ................................................................... ❑

2- أي من أنواع الطب النبوي التالية استخدمتها خلال الفترة السابقة؟

❑ الرقية الشرعية ❑ الحجامة ❑ العسل ❑ الحبة السوداء ❑ الحمية الغذائية

❑ غير ذلك، اذكره ..................................................................

3- هل استخدمت أي نوع من المكملات الغذائيه؟❑ نعم ❑ لا

- إذا كانت إجابتك عن السؤال السابق بنعم ,اذكر نوع العلاج الذي استخدمته خلال الشهر السابق ...............................

4- هل تستخدم اعشاب طبية في علاج حالتك الصحية ؟ اذكرها

..............................................................................................................................................

5- هل استخدمت أي نوع من تقنيات العقل والبدن ؟❑ نعم ❑ لا

أ - إذا كانت إجابتك عن السؤال السابق بنعم ,اذكر نوع العلاج الذي استخدمته خلال الشهر السابق

❑ التأمل ❑ الاسترخاء ❑اليوغا ❑ تمارين التنفس العميق ❑ تمارين الطاقة

-غير ذلك ,اذكره ...................................................................
